# The antimycotic 5-fluorocytosine is a virulence inhibitor of uropathogenic *Escherichia coli* and eradicates biofilm-embedded bacteria synergizing with β-lactams

**DOI:** 10.1128/aac.00280-25

**Published:** 2025-04-03

**Authors:** Srikanth Ravishankar, Antonietta Lucia Conte, Stacy Julisa Carrasco Aliaga, Valerio Baldelli, Karen Leth Nielsen, Moira Paroni, Maria Pia Conte, Paolo Landini, Elio Rossi

**Affiliations:** 1 Department of Biosciences, University of Milan 98853, Milan, Lombardy, Italy; 2 Department of Public Health and Infectious Diseases, Sapienza University 119653 , Rome, Lazio, Italy; 3 Department of Clinical Microbiology, Rigshospitalet 683301 , Copenhagen, Denmark; University of Fribourg, Fribourg, Switzerland

**Keywords:** uropathogenic *Escherichia coli*, urinary tract infections, biofilm, virulence, combination antimicrobial therapy

## Abstract

Biofilm can enhance antibiotic tolerance in bacteria, making treatment of biofilm-associated infections in clinical settings a significant challenge. 5-Fluorocytosine (5-FC), an FDA-approved drug mostly used as an antifungal, can hinder biofilm formation and production of virulence factors in Gram-negative bacteria. In this study, we tested 5-FC on nine uropathogenic *Escherichia coli* (UPEC) strains plus a fecal isolate. Our data indicated that 5-FC reduced curli fiber gene expression and inhibited virulence factors in UPEC strains. Unlike what was observed in other microorganisms, 5-FC antivirulence and antibiofilm properties were unaffected by either growth temperature or the medium pH, which might prove critical in urinary tract infection (UTI) treatment. Additionally, 5-FC impaired the expression of various UPEC virulence factors, including secreted toxins and type I and P fimbriae, thus leading to decreased UPEC adherence to bladder epithelial cells and improved survival of host cells. Finally, we found that a combination of 5-FC with β-lactams, but not other classes of antibiotics, significantly lowered the viability of bacteria in preformed biofilms. Despite a small set of pathogenic *E. coli* strains and an *in vitro* infection model, our findings strongly suggest that 5-FC might be a possible candidate as an antivirulence agent, particularly in a synergistic approach with β-lactam antibiotics.

## INTRODUCTION

Urinary tract infections (UTIs) are a widespread public health concern both in community and hospital settings (1). *Escherichia coli* is the leading pathogen behind UTIs worldwide (2). Notably, uropathogenic *E. coli* (UPEC) is the most frequent type of *E. coli* that can infect tissues outside the intestines (ExPEC) (3).

The success of UPEC in establishing the infection depends on the production of several virulence determinants. Adhesins, such as curli fibers (4), type-1 fimbriae (5), and P-fimbriae (6), contribute significantly to UPEC’s ability to cause UTIs. These adhesion factors are essential to form intracellular bacterial communities (IBCs) within the urinary tract epithelial cells. IBCs allow bacteria to evade the immune system and multiply undetected inside bladder cells, contributing to the persistence and recurrence of UTIs (7). In addition, UPECs can secrete soluble toxins, such as exotoxin α-hemolysin (*hlyA*), that promote red blood cell lysis and cytotoxic damage to the host uroepithelium (8). Similarly, secreted toxins termed Serine Protease Autotransporters of *Enterobacteriaceae* encoded by genes like *sat, pic,* and *vat* have immunomodulatory activity, stimulate vacuole formation, and lead to urothelial barrier dysfunction in both bladder and kidneys (9–11).

Antibiotic resistance is another crucial aspect of UPEC infections, particularly associated with recurrent UTIs (12). Antimicrobial resistance and recalcitrant infection are further exacerbated by the formation of bacterial biofilms (13). Unlike planktonic bacteria, biofilms are dense, diverse, and organized microbial communities (14, 15) encased in self-made extracellular polymeric substances (EPS). As biofilms, bacteria exhibit a remarkable resilience against most antibiotics used in therapy (16, 17), and their treatment poses a significant challenge. For example, transition to a slow-growing or dormant state within biofilms reduces the susceptibility to antibiotics that target cell division (18). Furthermore, biofilm EPS might hinder the penetration of antimicrobials and modulate bacterial cell recognition by the immune system (19).

Several studies have shown that the FDA-approved antimycotic drug 5-fluorocytosine (5-FC) and other fluoropyrimidines are endowed with antibiofilm activity against several bacteria (20, 21); recently, we have shown that 5-FC antibiofilm activity is mediated at least in part by inhibition of *de novo* pyrimidine synthesis in the *E. coli* reference strain MG1655 (22). In this work, we show that 5-FC is also active against clinically relevant UPEC isolates obtained from uncomplicated UTIs. Furthermore, we showed that the biological activity of 5-FC is not restricted to biofilm inhibition. This nucleobase analog acts as a potent virulence factor inhibitor in UPEC, impairing the expression of several virulence determinants, hampering hemolysis, and reducing bacterial adhesion and cytotoxicity against bladder epithelial cells. Additionally, we show that 5-FC combination with β-lactams, but not other classes of antibiotics, results in strong eradication of bacteria within pre-formed, mature biofilms.

## RESULTS

### 5-Fluorocytosine prevents UPEC biofilm formation via pyrimidine starvation and curli fiber inhibition

**Fig 1:**
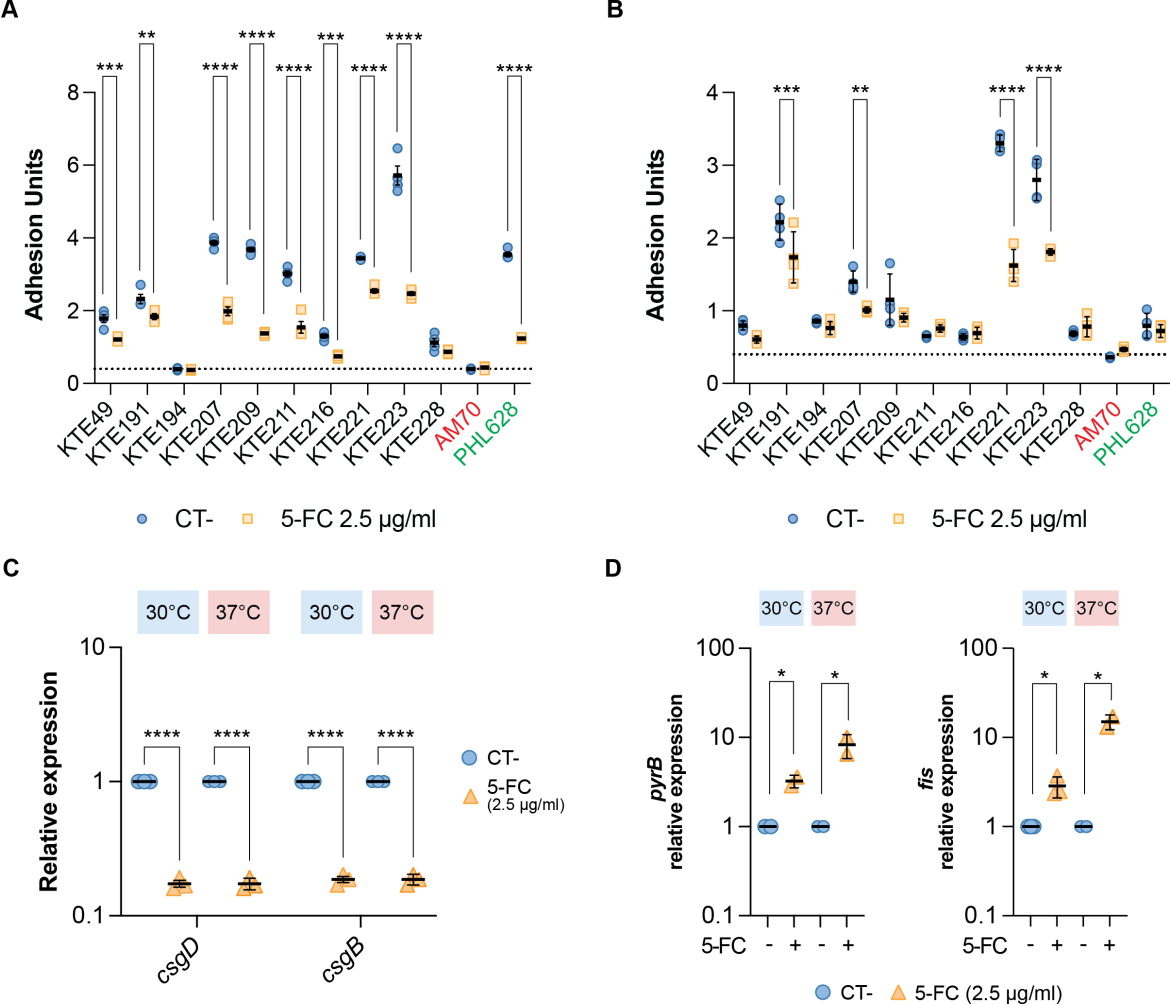
5-FC effect on biofilm formation, curli expression, and pyrimidine nucleotide pool in UPEC strains. Bacterial adhesion at (**A**) 30°C and (**B**) 37°C. The assays were performed on 9 UPEC clinical isolates and 1 fecal isolate (KTE49) in the presence or absence of 2.5 µg/mL 5-FC. Relative expression of (**C**) curli biosynthetic genes *csgD, csgB,* and (**D**) pyrimidine nucleotide pool-responsive genes *pyrB and fis* was determined by quantitative real-time PCR (RT-qPCR) analysis on RNA extracted from he UPEC strain KTE223 in the presence or absence of 2.5 µg/mL 5-FC at 30°C and 37°C. In panels A and B, the dotted line represents the average adhesion of the curli-deficient AM70 strain. In panels C and D, values are expressed as relative units, setting the untreated control to 1. Results of at least three independent replicates, mean, and standard deviation are shown in dot plots. *, *P-*value < 0.05; **, *P*-value < 0.01; ***, *P*-value < 0.001; ****, *P*-value < 0.0001, two-way analysis of variance (ANOVA) with Dunnett’s test for multiple comparisons.

In the reference strain MG1655, 5-FC inhibits biofilm formation via transcription inhibition of genes encoding curli fibers (22). Curli are important fitness factors in UPEC strains (4, 23). Thus, we evaluated whether 5-FC could also display antibiofilm activity against UPEC, testing biofilm formation and adhesion on 10 clinical isolates (nine UPEC and one fecal isolate) after treatment with 5-FC. Experiments were performed at 30°C, at which curli expression is at its maximum in laboratory conditions (24), and at 37°C, i.e*.,* the standard host temperature. 5-FC was able to cause a 1- to 2.3-fold reduction in the biofilm adhesion of three-quarters of the tested UPEC clinical isolates at both 30°C (Fig. 1A) and 37°C (Fig. 1B) at 2.5 µg/mL, a subinhibitory concentration for *E. coli* (22).

Given the significant variability in urine pH (25) and reports that 5-FC antimicrobial activity is influenced by pH in another microorganism (26), we assessed its antimicrobial and antibiofilm properties across different pH conditions using specific clinical isolates. Notably, we observed no inhibitory effect on bacterial growth up to the highest 5-FC concentration tested (256 µg/mL) (Fig. S1), in contrast to the laboratory strain MG1655, in which 32 µg/mL 5-FC resulted in a 50% growth reduction (22). Similarly, at all tested pH levels, 5-FC at 2.5 µg/mL, i.e., well below an inhibitory concentration for growth, under all conditions, consistently inhibited biofilm formation at 30°C (Fig. S1B) and 37°C (Fig. S1C). While minor variations in fold reduction were observed, these fluctuations were not attributable to specific pH, strain, or temperature, suggesting they resulted from stochastic variations rather than a systematic effect of pH.

Overall, these findings indicate that pH does not alter the antimicrobial and antibiofilm properties of 5-FC across a broad range of values. Therefore, for all subsequent experiments, we used the standard YESCA medium at pH 6.4 ± 0.2, which falls within the median urinary pH range for both males and females (27).

In the reference strain MG1655, 5-FC-dependent adhesion inhibition is due to the downregulation of curli-encoding *csgBAC* and *csgDEFG* operons at the transcription level, in turn, as a response to inhibition of *de novo* synthesis and the pyrimidine starvation induced by 5-FC (22). By focusing on the KTE223 isolate, a strong biofilm producer (Fig. 1A and B), we could observe that 5-FC treatment inhibited expression of *csgB* and *csgD* genes both at 30°C and 37°C by 5.3- and 5.7-fold, respectively (Fig. 1C). In addition, 5-FC exposure stimulated the expression of *de novo* pyrimidine pathway gene *pyrB* (3.2-fold at 30°C and 8.3-fold at 37°C) and nucleotide-responsive regulator Fis (2.8-fold at 30°C and 15-fold at 37°C) (Fig. 1D), whose expression is activated in response to a decrease in the pyrimidine pool, strongly suggesting that 5-FC treatment leads to pyrimidine starvation in UPEC, similar to what is observed in *E. coli* MG1655 (22).

### 5-Fluorocytosine inhibits the expression of virulence determinants hindering UPEC pathogenicity

**Fig 2:**
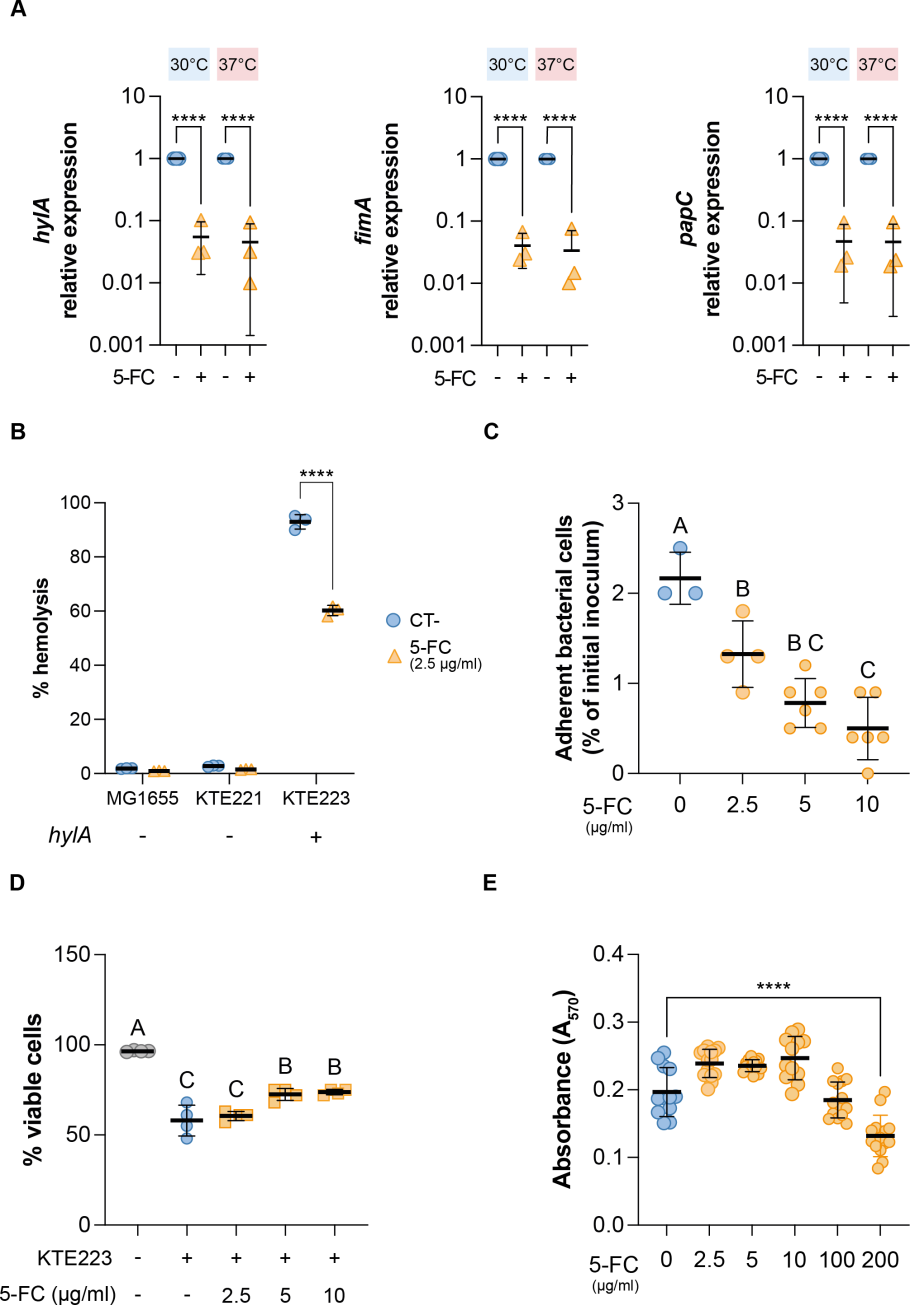
5-FC effect on the expression of virulence factors and pathogenesis of UPEC. (**A**) Relative expression of virulence determinant *fimA, hlyA,* and *papC* determined by RT-qPCR analysis on RNA extracted from KTE223 strain in the presence or absence of 2.5 µg/mL 5-FC at 30°C and 37°C. The values are expressed as relative units, setting the untreated control to 1. (**B**) Hemolysis activity of *hlyA*-negative (MG1655 and KTE221) and *hlyA*-positive (KTE223) *E. coli* strains with or without 2.5 µg/mL 5-FC. (**C**) KTE223 adhesion to bladder epithelial cells in the presence or absence of increasing concentrations of 5-FC. (**D**) Epithelial cell viability during infection with or without KTE223 and increasing concentrations of 5-FC. Results from the trypan blue exclusion assay are shown. (**E**) Cytotoxic effect of increasing concentrations of 5-FC on bladder epithelial cells after 24 hours of incubation. Results from the colorimetric MTT assay are shown. Results of at least three independent replicates, mean, and standard deviation are shown in all dot plots. *, *P-*value < 0.05; **, *P*-value < 0.01; ***, *P*-value < 0.001; ****, *P*-value < 0.0001 (two-way ANOVA with Dunnett’s test for multiple comparisons). Letters indicate significant within-group differences between treatments (one-way ANOVA with Tukey’s test for multiple comparisons).

In addition to inhibition of curli production, *de novo* pyrimidine biosynthesis perturbation affects type I fimbriae expression in adherent-invasive *Escherichia coli* (28). Therefore, we reasoned that exposure to 5-FC might also negatively impact the production of other virulence factors in UPEC. Thus, we tested the expression of type I fimbriae gene *fimA* and several other virulence determinants encoded in the genome of the KTE223 strain, namely, P fimbriae (*papC*), α-hemolysin (*hlyA*), and secreted autotransporter toxins (*pic, vat, sat*, and *sinH*) upon 5-FC treatment (Fig. 2A; Fig. S2).

5-FC was able to reduce the expression of the adhesin-encoding genes *fimA* and *papC* by 30 and 22 times, respectively, at host temperature (Fig. 2A). Similarly, we observed repression of secreted toxin-encoding genes (Fig. 2A; Fig. S2): *hlyA, pic,* and *sinH* expression was reduced 22-, 3.6-, and 2.6-fold, respectively, at 37°C, while *vat* and *sat* genes showed repression (3.2- and 3.8-fold, respectively) only at 30°C. Consistent with the inhibition of *hlyA* expression, the KTE223 strain showed a 33% reduction in its hemolytic activity when treated with 5-FC at 2.5 µg/mL (Fig. 2B). Very slight, although nonsignificant, effects were also observed in poorly hemolytic *hlyA*-negative strains KTE221 and MG1655, used as negative controls in the experiment (Fig. 2B).

Due to its strong antivirulence activity, we tested the possible effects of 5-FC on KTE223 bacterial adhesion to epithelial bladder cells. As shown in Fig. 2C, 5-FC reduced KTE223 adhesion in a dose-dependent manner by up to 4.3 times, consistent with inhibition of adhesion factor production (Fig. 2A).

In contrast, despite a reduction in KTE223 adhesion in infection experiments, survival of epithelial cells was only slightly increased by 5-FC (Fig. 2D), possibly due to residual production of soluble exotoxins by KTE223. However, epithelial cell survival did increase from 55% up to 74% in the presence of 10 µg/mL 5-FC, a dose well below toxic concentrations for bladder epithelial cells (Fig. 2E) or the bacterium (Fig. S1A). Overall, our data indicate that 5-FC activity is not restricted to the inhibition of curli gene expression but can generally affect the production of several KTE223 virulence determinants. Indeed, although not fully proficient in counteracting the KTE223 cytotoxic effect (Fig. 2D), 5-FC might have some potential to modulate its cytotoxicity during colonization and acute infection.

### 5-FC potentiates β-lactam antimicrobial activity against KTE223 biofilm

**Fig 3:**
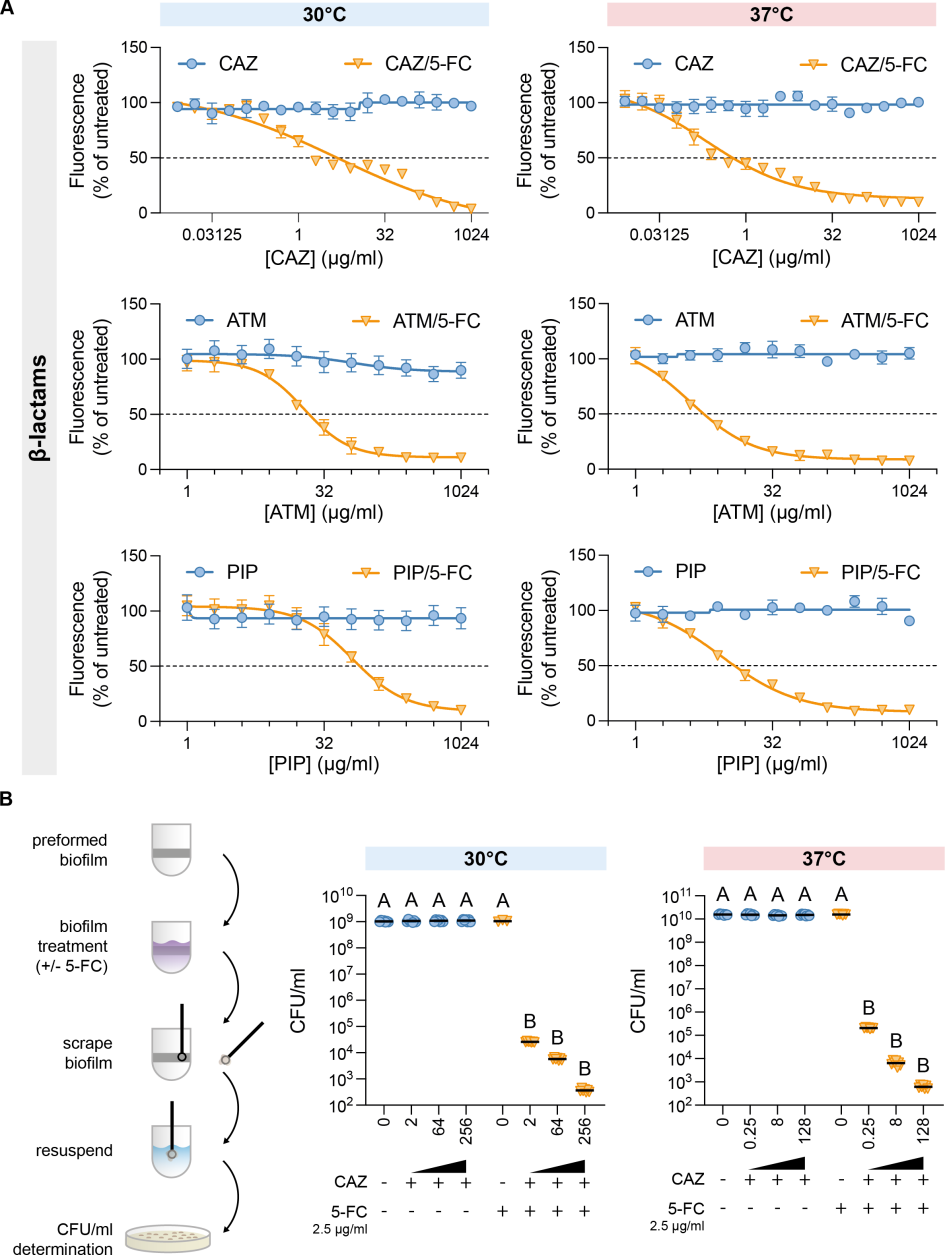
5-FC synergy with β-lactams in a mature biofilm of the KTE223 strain. (**A**) Metabolically active bacterial cells residing in the mature biofilm of KTE223 strain untreated or treated with increasing concentrations of β-lactams (CAZ, ATM, and PIP) and their combination with 2.5 µg/mL 5-FC at 30°C and 37°C was determined using FDA assay. Fluorescence emitted by the untreated mature biofilm of the KTE223 strain is considered 100%. (**B**) Colony-forming units per milliliter (CFU/mL) were determined by agar plating method in scrapped biofilm after treating KTE223 mature biofilm with select concentrations of CAZ and its combination with 2.5 µg/mL 5-FC at 30°C and 37°C. The scheme on the left represents the methodology followed to perform this experiment. Results of 3–7 independent replicates and standard deviations are shown. Results of at least three independent replicates, mean, and standard deviation are shown in dot plots. Letters indicate significant within-group differences between treatments (one-way ANOVA with Tukey’s multiple comparisons test).

While 5-FC can prevent *E. coli* biofilm formation and adhesion, the main challenge in clinical settings is the treatment of preformed mature biofilms, often recalcitrant to antibiotics (13). Thus, using the strong biofilm-producing UPEC strain KTE223, we evaluated the ability of 5-FC to eradicate preformed biofilms and affect bacterial viability within the community. Furthermore, we tested its potential effect when combined with different antibiotics used to treat UTIs (29). To this end, we measured the minimum biofilm eradication concentration (MBEC) of 5-FC alone at 2.5 µg/mL, i.e*.*, the sub-inhibitory concentration able to reduce adhesion (Fig. 1A and [22]), and in combination with increasing concentrations of ciprofloxacin, gentamicin, ceftazidime (CAZ), piperacillin (PIP), and aztreonam (ATM). 5-FC treatment and its combination with antibiotics did not disrupt the mature biofilm formed by KTE223 at all concentrations tested, always showing the same amount of adherent cells as the untreated control when measured with the crystal violet method (Fig. S3 and S4). However, when we evaluated bacterial viability within the biofilm using fluorescein diacetate (FDA), we observed that, while the single use of any antibiotics did not result in a reduction of bacterial viability (Fig. 3A; Fig. S5), the combination of 5-FC with β-lactams (CAZ, ATM, PIP) caused a substantial decrease in FDA fluorescence (Fig. 3A). For instance, the combination of 2.5 µg/mL 5-FC with CAZ caused a 50% reduction of fluorescence at 2 µg/mL. This effect was similar at 30°C and 37°C (Fig. 3A), with a slightly higher impact at host temperature. Consistently, the enumeration of living cells within the preformed biofilm after treatment (Fig. 3B) indicated a 50% reduction in CFU/mL upon combining 2.5 µg/mL 5-FC with 2 µg/mL CAZ. We did not observe any viability reduction either with 5-FC alone (Fig. S5A) or combined with fluoroquinolones or aminoglycosides (Fig. S5B), thus suggesting a specific synergistic mechanism with β-lactams. This effect was confirmed in other biofilm-forming isolates, such as KTE191 and KTE221 (Fig. S6). Remarkably, bacterial titer in supernatants used for treating mature biofilm showed no differences in CFU/mL in the presence of either 5-FC or 5-FC with different CAZ concentrations (Fig. S7), indicating that biofilm-embedded bacteria are not released into spent media after the drug treatment and that the reduction of viability is indeed due to bacterial cell death within the biofilm structure.

Overall, our findings demonstrate that 5-FC exhibits specific synergy with β-lactams, effectively eliminating bacteria within biofilms. This is particularly significant given that biofilm-associated microorganisms are inherently resistant to conventional treatments without physically disrupting the biofilm structure.

## DISCUSSION

The significant healthcare challenge posed by bacterial biofilm largely depends on their resistance to antibiotics, rendering current treatments largely ineffective (16, 17). Biofilm-mediated antibiotic tolerance leads to relapsing infections, such as those observed in recurrent UTIs (13). Despite extensive research on the identification of new antibiofilm drugs (30, 31), no major new classes of antimicrobials or antivirulence drugs have made their way into clinical therapy.

In this work, we confirmed previous evidence that 5-FC antibiofilm properties against laboratory *E. coli* strain MG1655 (22) can also be observed against clinically relevant *E. coli* strains isolated mainly from UTI. The reduction in biofilm formation by 5-FC not only takes place at 30°C, the optimal temperature for curli fiber production and biofilm formation in laboratory conditions, but also at 37°C (Fig. 1A and B), and across a broad pH range (from 8 to 5), which encompasses the physiological and pathological variations in urine pH (Fig. S1). These findings support the drug’s potential applicability in physiologically relevant conditions. Additionally, pH variations did not influence the minimum inhibitory concentration (MIC) of 5-FC in clinical isolates of *E. coli*, contrasting with previous findings in *Aspergillus fumigatus* (26) and highlighting the differences in physiology between the two distant organisms. Notably, clinical isolates exhibited no detectable MIC for 5-FC up to 256 µg/mL, well above the concentration necessary to inhibit the laboratory strain MG1655 (22). This observation has interesting implications: the clinical isolates tested showed greater physiological resistance to 5-FC as an antimicrobial, and the concentrations employed in our study (2.5, 5.0, 10 µg/mL) were well below the inhibitory threshold, minimizing selective pressure for resistance while effectively reducing the pathogenic potential of the tested strains.

5-FC’s biological activity was not limited to biofilm inhibition. The molecule exerted a broader clinically relevant effect by reducing, albeit to different extents, the expression of several virulence factors conserved in UPEC. This included adhesins such as curli fibers, type-1 fimbriae, and P-fimbriae that are involved in colonizing host cells, as well as α-hemolysin and secreted toxins (Pic, Vac, Sat, SinH) that cause damage to host tissues (8–11). Although KTE223 pathogenicity was not totally obliterated, low levels of 5-FC were sufficient to reduce bacterial colonization of the bladder epithelium and led to a slight increase in survival of host cells upon KTE223 infection (Fig. 2C and D). Lack of complete abrogation of secreted toxins might be responsible for the relatively modest increase in epithelial cell viability, despite strong reduction in cell adhesion (Fig. 2C and D). This suggests that, although the introduction of 5-FC in clinical practice as an antivirulence drug might not be totally effective, it could find better use as an adjuvant to antibiotic treatment. Indeed, 5-FC only seems capable of preventing colonization but cannot disrupt preformed biofilm, a condition that often needs to be addressed in clinical practice (13). Biofilms reduce antibiotic efficacy, and the number of molecules that can directly target bacteria within a biofilm is scarce (32). In this context, 5-FC shows a peculiar and attractive feature. Indeed, 5-FC was able to potentiate the antibacterial activity of the peptidoglycan biosynthesis-targeting classes of antibiotics (β-lactams/cephalosporins) against *in vitro*-grown biofilm-embedded UPEC (Fig. 3; Fig. S6). The effect is specific, and 5-FC showed no synergy with ciprofloxacin and gentamicin, which target DNA replication and protein synthesis, respectively (Fig. S5). Although the mechanism of action is currently unknown, 5-FC exhibits antibiofilm activity by affecting intracellular pyrimidine availability in the *E. coli* laboratory strain MG1655, partly via a major peptidoglycan synthase enzyme (22). Increased expression of genes responding to pyrimidine nucleotide availability upon 5-FC treatment (Fig. 1D) in UPEC and the observation that 5-FC synergizes with drugs targeting peptidoglycan biosynthesis further suggest the existence of a conserved connection between pyrimidine nucleotide biosynthesis and virulence mechanisms in *E. coli* pathotypes*,* which would not be restricted to UPEC but also apply to other pathogenic strains, as we recently showed (28).

Since 5-FC is already used in clinical practice, concerns about bioavailability and safety are low, consistent with our cytotoxicity assay results (Fig. 2E). Therefore, repurposing 5-FC could serve as an innovative approach to meet the clinical need for effective treatments against biofilm-based infections (33). This could involve combining it with β-lactams to eradicate biofilm bacteria or, in some instances, using it alone to minimize tissue damage and prevent reinfection by inhibiting virulence factors. Thus, despite the limitations of our study, such as the small number of clinical isolates tested and the lack of *in vivo* infection models, we have good evidence, even from previous literature, that inhibition of pyrimidine biosynthesis, whether through genetic or chemical means, influences biofilm formation and virulence in different *E. coli* pathotypes (28). In particular, the synergy between 5-FC treatment and β-lactams, likely connected to perturbations in cell wall synthesis by 5-FC (22), appears to be a promising approach for the treatment of biofilm infection and suggests that novel inhibitors of pyrimidine *de novo* biosynthesis might have similar, or even better potential.

## MATERIALS AND METHODS

### Bacterial strains and growth conditions

The bacterial strains used in this study and their corresponding epidemiological and clinical data are listed in Table S1. The study included nine UPEC clinical isolates and one *E. coli* isolate from the fecal flora of a healthy control, previously described in the literature (34). UPEC isolates were collected from the Department of Clinical Microbiology at Rigshospitalet, Denmark, and were derived from patients with UTIs, including both females (approximately 78%) and males (approximately 22%). The infections were classified as 11% hospital-acquired and 88% community-acquired. The isolates were selected to represent diverse UPEC genotypes, distributed among phylogenetic groups A (11%), B2 (77%), and D (11%). Antibiotic susceptibility testing revealed that all isolates were susceptible to ampicillin, cefpodoxime, ceftiofur, chloramphenicol, ciprofloxacin, gentamicin, nalidixic acid, sulfamethoxazole, tetracycline, and trimethoprim, except KTE223, which was resistant to ampicillin and sulfamethoxazole. Furthermore, the isolates were included based on a positive screening for the presence of the curli-encoding operons (*csgBAC* and *csgGDEF*).

For the assays, bacteria were routinely grown in YESCA medium (10 g/L casamino acids, 1.5 g/L yeast extract, 0.05 g/L magnesium sulfate, 0.005 g/L manganese chloride) at 30°C or 37°C in shaking conditions (150 rpm) or LB agar medium (10 g/L tryptone, 5 g/L yeast extract, 5 g/L sodium chloride, 15 g/L agar). YESCA pH was determined for several batches and was 6.4 ± 0.2. To test the effect of nucleobase analog, 5-fluorocytosine (5-FC; stock concentration: 10 mg/mL in sterile milli-Q H_2_O) was added at specified concentrations. The impact of pH on 5-FC activity was assessed by adjusting the medium to the desired pH values using NaOH or HCl.

### Gene expression analysis

Gene expression levels were measured using quantitative real-time PCR (RT-qPCR), as previously described (22). After 24 hours of incubation, RNA was extracted from cultures grown in YESCA medium with or without 5-FC at 30°C or 37°C in shaking. The complete list of primers used for amplification is reported in Table S2.

### Biofilm formation

For static biofilm adhesion assays, overnight *E. coli* cultures grown in YESCA medium were normalized to OD_600_ = 0.025 and incubated in the YESCA medium in a 96-well flat bottom plate for 24 hours at 30°C or 37°C in the static condition. Adhesion was determined using the Crystal Violet (CV) staining method, as previously reported (22).

### Minimum Biofilm Eradication Concentration (MBEC) and bacterial viability determination in mature biofilm

The MBEC of 5-FC and its combination with antibiotics on preformed *E. coli* biofilm was evaluated using the standard microdilution method, according to the Clinical and Laboratory Standards Institute guidelines with minor changes (35). Briefly, overnight cultures were normalized in 200 µL of YESCA to an OD_600_ ≈ 0.025 in 96-well microtiter plates and then incubated at 30°C or 37°C in static conditions to allow biofilm formation. After 20 hours of growth, the planktonic cells were removed and transferred to a new plate for the OD_600_ reading. The pre-formed biofilm was treated with 200 µL of YESCA containing the desired dilutions of 5-FC, antibiotics, or their combinations. The plates were incubated at 30°C or 37°C for 6 hours. The spent media were removed, and biofilm quantification was performed using the CV staining method as described earlier. Simultaneously, the same procedure was repeated to determine the viability and metabolic activity of bacteria inside preformed biofilm by measuring the hydrolysis of FDA to yellow fluorescent molecule “fluorescein” according to reference 36. Briefly, a solution of 10 mg/mL FDA in acetone was diluted to a final concentration of 0.1 mg/mL in 100 mM 3-(N-morpholino)-propane sulfonic acid (MOPS, pH 7.0), and 200 µL of this solution was added to wells with drug-treated or untreated pre-formed biofilm after removing the spent media. After 1 hour of incubation at 37°C in static conditions, the absorbance is read at 494 nm, and the fluorescence is expressed as “% of untreated,” a function of untreated condition (100%).

For CFU determination in preformed biofilm after treatments, biofilm is scrapped using a sterile inoculation loop and resuspended thoroughly in YESCA medium by pipetting vigorously after removing the spent media from the wells. The spent media and the biofilm resuspension are serially diluted and plated on LB agar plates. After overnight incubation at 37°C, colonies were counted, and bacterial titer was expressed as CFU per milliliter (CFU/mL). Untreated pre-formed biofilm was used as a negative control.

### Hemolysis activity assay

The hemolytic activity was determined as previously described (37, 38) with minor modifications. The overnight-grown *E. coli* cultures were centrifuged at 5,000 rpm for 10 minutes and resuspended in YESCA medium to reach an OD_600_ of 0.2. The cultures (~10^7^ CFU/mL) were then incubated with 5% (final concentration) defibrinated sheep blood and 10 mM CaCl_2_ at 37°C in a total volume of 1 mL. A 9 g/L NaCl was added to all samples to prevent hypo-osmotic lysis of the erythrocytes. After 2 hours of incubation at 37°C with gentle agitation (300 rpm), intact erythrocytes were harvested by centrifugation (10,000 rpm) at 4°C for 8 minutes. Hemoglobin released in supernatants was determined by measuring the absorbance at 545 nm wavelength (A545). The % hemolysis (P) was calculated using the equation P = [(X − B)/(T − B)] × 100, where X is the A545 of the sample analyzed, while B and T represent the baseline and total hemolysis*,* i.e*.,* the A545 obtained with sterile YESCA (10 mM CaCl_2_ and 9 g/L NaCl added) and double-distilled water, respectively.

### Cell line cultivation

The human bladder cancer T24 cell line (ATCC HTB-4) was cultured in Roswell Park Memorial Institute 1640 medium supplemented with 10% heat-inactivated fetal bovine serum (FBS; SAFC Biosciences Inc., Lenexa, KS, USA), 100 IU/L penicillin, and 100 mg/L streptomycin. Cultures were maintained in a humidified atmosphere containing 5% CO_2_ at 37°C.

### Adhesion to T24 cell monolayers

The adhesiveness of *E. coli* strain KTE223 to T24 monolayers was assayed by culturing the cells in a 24‐well plate at a density of 2 × 10^5^ cells/mL for 24 hours at 37°C in 5% CO_2_. *E. coli* KTE223 was grown in YESCA with or without 5-FC at 37°C. After overnight growth, bacteria were sub-cultured (1:100) in a fresh medium with or without 5-FC for 2 hours at 37°C to obtain exponentially grown bacteria.

T24 cells were infected with bacteria in the presence or absence of 5-FC in the cell medium, at a multiplicity of infection of approximately one bacterium per cell. They were then centrifuged twice at 500 × *g* for 2.5 minutes to synchronize infection and incubated for 30 minutes at 37°C in 5% CO_2_. After incubation, cells were extensively washed with PBS to remove unattached bacteria, lysed, added ice‐cold 0.1% Triton X‐100, and seeded on TSA plates to obtain the total viable count. Bacteria were considered adherent when the mean adhesion index (no. of adherent bacteria/initial inoculum) was ≥0.8%.

### Epithelial cell survival determination by trypan blue assay

T24 cell viability, with or without *E. coli* KTE223 and 5-FC, was determined 30 minutes post-infection. Cells were detached by trypsinization, and the number of viable cells in each experimental condition was counted using a trypan blue solution (Sigma-Aldrich, Milan, Italy). One part of 0.4% trypan blue was mixed with one part of cell suspension. The mixture was incubated for ∼3 minutes at room temperature. Then, 10 µL of the trypan blue:cell mixture was added to a hemacytometer. The number of unstained (viable) and stained (nonviable) cells was determined. The viability of the control (untreated cells) was regarded as >95%. The percentage of viable cells was calculated as follows:


$$\frac{total\;number\;of\;viable\;cells\;per\;ml\;of\;aliquot}{total\;number\;of\;cells\;per\;ml\;of\;aliquot}\times 100$$

### Cytotoxicity studies by MTT assay

T24 cells at 5 × 10^5^ cells/mL concentration were seeded in 96-well plates and cultured for 24 hours at 37°C with 5% CO_2_. Three concentrations of 5-FC (2.5, 5, and 10 µg/mL) were added to cell monolayers and incubated for 30 minutes. Then, 100 µL of 0.5 mg/mL of 3-(4,5-dimethylthiazol-2-yl)-2,5-diphenyltetrazolium bromide (MTT) reagent was added to each well, and plates were incubated at 37°C for 4 hours. Afterward, the dye was eluted with 200 µL of DMSO for 10 minutes at room temperature, and the optical density at 568 nm was measured using a microplate reader (PerkinElmer, Boston, MA, USA). The viability percentage (%) was calculated as mean OD treatment/mean OD control × 100.

## SUPPLEMENTAL MATERIAL

Supplemental material aac.00280-25-s0001.pdf Figures S1 to S7; Tables S1 and S2.

ASM does not own the copyrights to Supplemental Material that may be linked to, or accessed through, an article. The authors have granted ASM a non-exclusive, world-wide license to publish the Supplemental Material files. Please contact the corresponding author directly for reuse.
