# Supplementary material for: The antimycotic 5-fluorocytosine is a virulence inhibitor of uropathogenic Escherichia coli and eradicates biofilm-embedded bacteria synergizing with β-lactams
Source: Antimicrob Agents Chemother. 2025 Apr 3;69(5):e00280-25. doi: 10.1128/aac.00280-25 (PMC12057335; doi:10.1128/aac.00280-25)
Supplement: Supplemental material — Figures S1 to S7; Tables S1 and S2. [file aac.00280-25-s0001.pdf]

Supplementary Information (SI)

The antimycotic 5-fluorocytosine is a virulence inhibitor of uropathogenic *Escherichia coli* and eradicates biofilm-embedded bacteria synergizing with β-lactams

Srikanth Ravishankar[a], Antonietta Lucia Conte[b], Stacy Julisa Carrasco Aliaga[a], Valerio Baldelli[a], Karen Leth Nielsen[c], Moira Paroni[a] , Maria Pia Conte[b], Paolo Landini[a, #], Elio Rossi[a, #]

[a] Department of Biosciences, University of Milan, Milan, Italy

[b] Department of Public Health and Infectious Diseases, Sapienza University, Rome, Italy

[c] Department of Clinical Microbiology, Rigshospitalet, Copenhagen Ø, Denmark

[#] **Corresponding authors:**

Dr. Elio Rossi, elio.rossi@unimi.it

Prof. Paolo Landini, paolo.landini@unimi.it

Table of contents

Supplementary Figures **3**

Figure S1 3

Figure S2 4

Figure S3 5

Figure S4 6

Figure S5 7

Figure S6 8

Figure S7 9

Supplementary Table 10

Table S1 10

Table S2 11

Supplementary references 12

Supplementary Figures

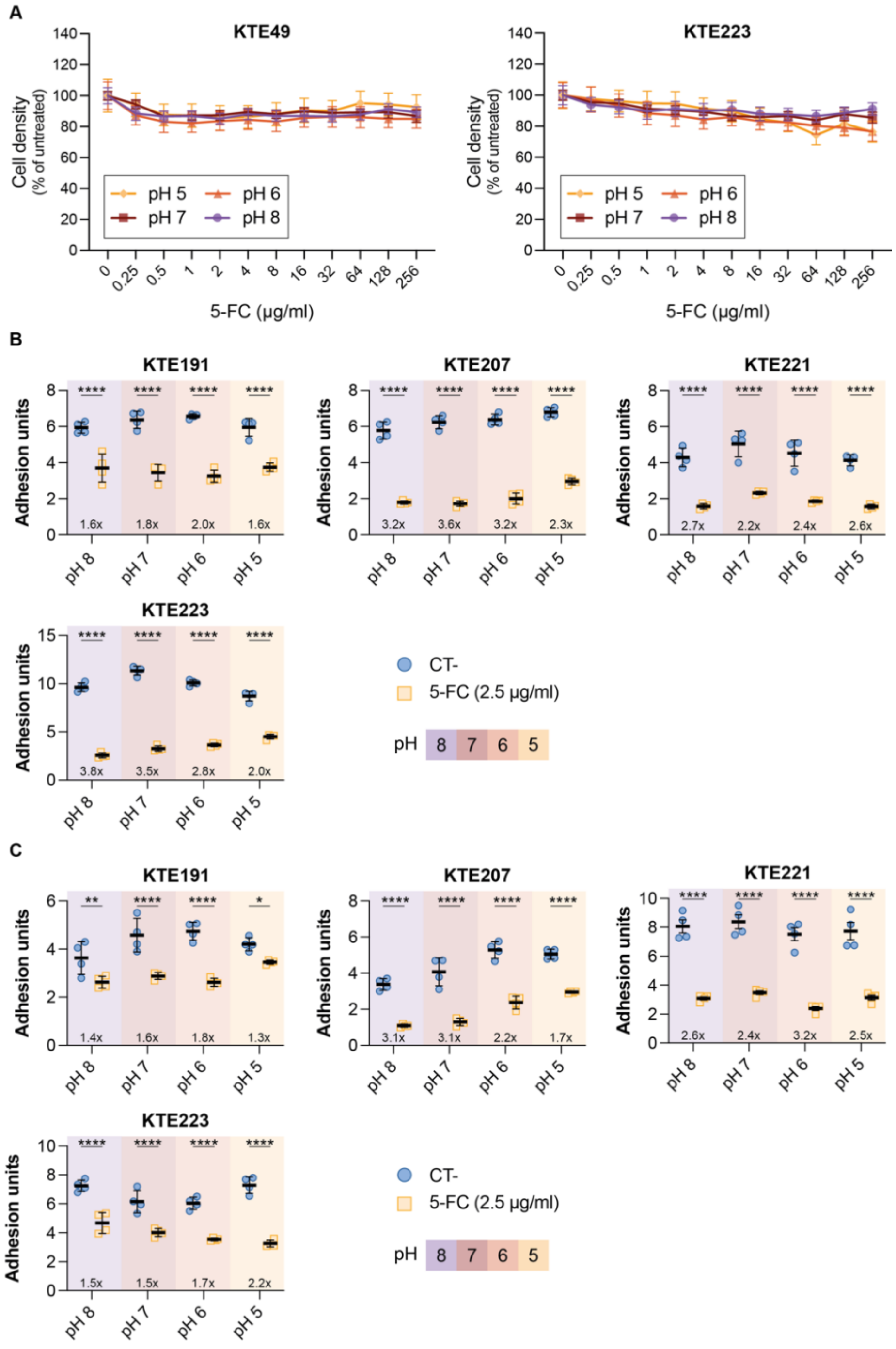

Figure S1. Effect of pH on 5-fluorocytosine (5-FC) antimicrobial and antibiofilm properties. (A) Effect of increasing concentrations of 5-FC on bacterial viability, expressed as changes in the percentage of cell density (OD_{600}) compared to the untreated conditions (0 µg/ml 5-FC, 100%) after 24 hours of growth at 37°C in YESCA medium at specific pH. Results represent the mean and standard deviation of 4 independent replicates. **(B, C)** Bacterial adhesion to polystyrene of strong biofilm-forming UPEC strain KTE191, KTE207, KTE221, KTE223 after 24 hours of growth in YESCA medium at specific pH in the presence or absence of 2.5 µg/ml 5-FC. Adhesion was assessed at 30°C (panel B) and 37°C (panel C). The results of 4 independent replicates, means, standard deviations and fold reduction between treated (5-FC) and untreated (CT-) are shown in dot plots. *, p-value < 0.05, ***, p-value < 0.001, ****, p-value < 0.0001, two-way ANOVA with Šidák correction for multiple comparisons.

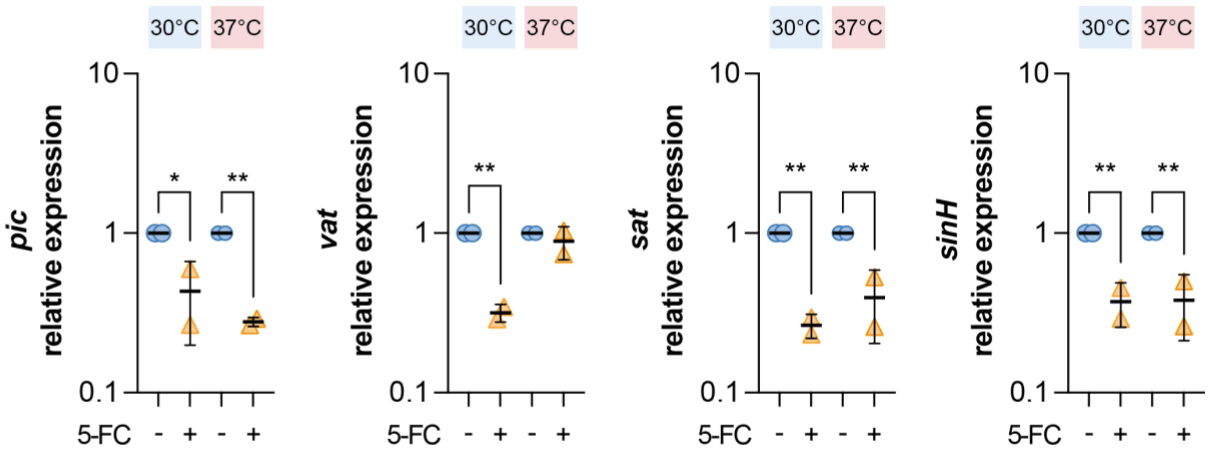

Figure S2. 5-fluorocytosine (5-FC) effect on transcription of autotransporter toxin genes in KTE223 strain. Relative expression of autotransporter toxin genes *pic, vat, sat*, and *sinH* determined by RT-qPCR analysis on RNA extracted from KTE223 strain in the presence (triangles) or absence (dots) of 2.5 µg/ml 5-FC at 30°C and 37°C. The values are expressed as relative units, setting the untreated control to 1. The results of 2 independent replicates, means, and standard deviations are shown in dot plots.. *, p-value < 0.05; **, p-value < 0.01, one-way ANOVA with Šidák correction for multiple comparisons.

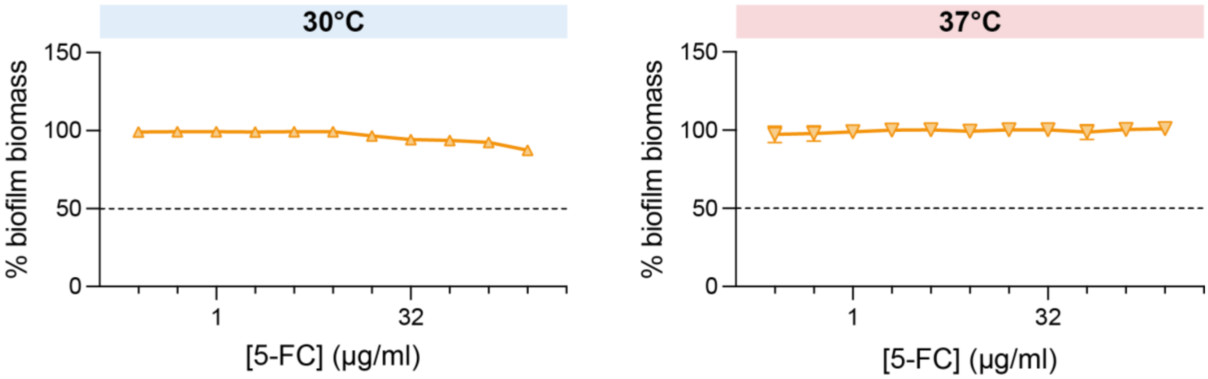

Figure S3. The effect of 5-fluorocytosine (5-FC) on the mature biofilm of the KTE223 strain. Percentage of residual preformed biofilm biomass of the KTE223 strain after exposure to increasing concentrations of 5-FC (5-fluorocytosine) at 30°C and 37°C, as determined via crystal violet (CV) staining. CV values in untreated samples were considered 100%. Results from two independent replicates and their standard deviations are shown.

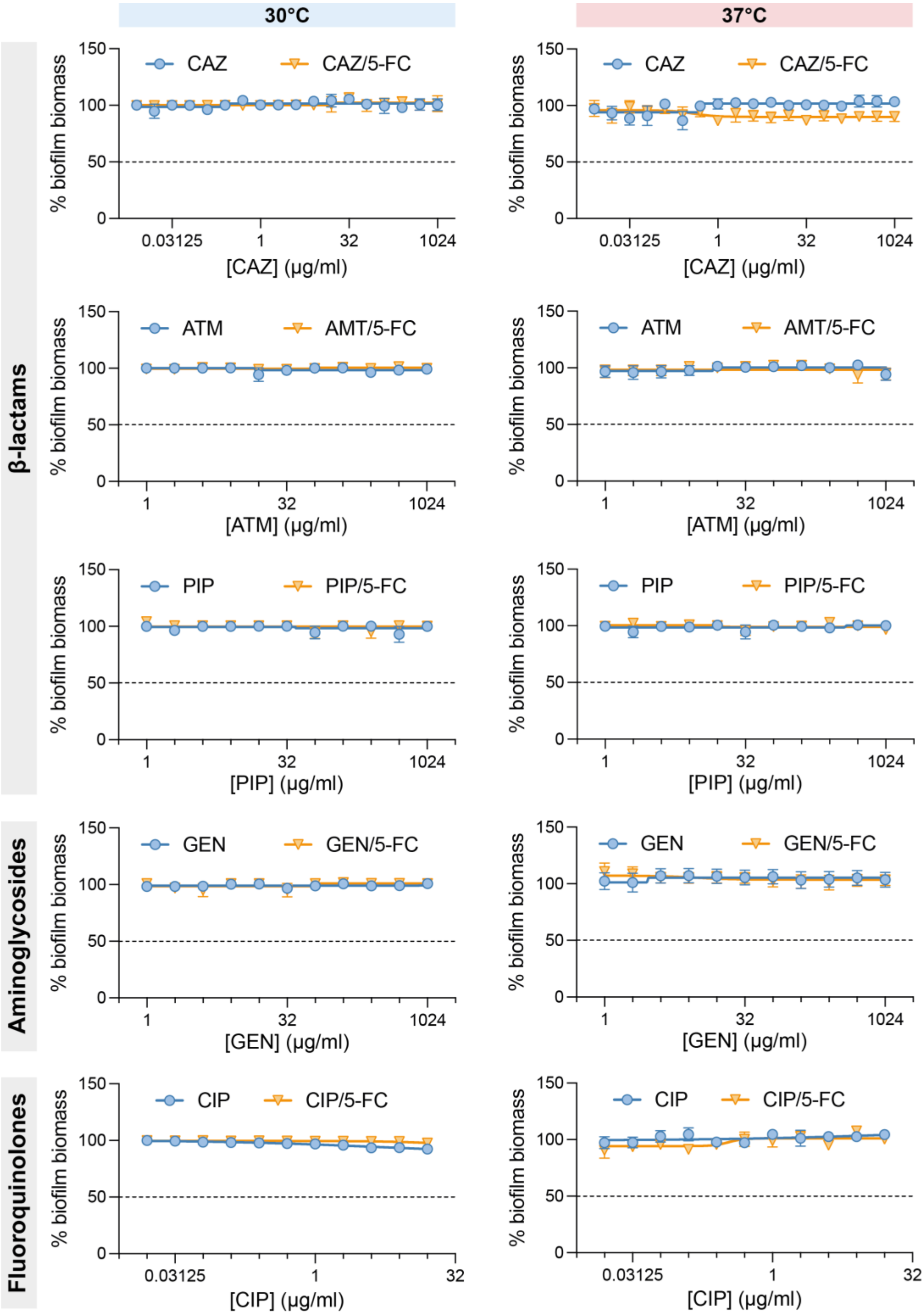

Figure S4. Effect of antibiotics and their combination with 5-fluorocytosine (5-FC) on mature biofilm of KTE223 strain. Biofilm biomass of 20-hour preformed biofilm of KTE223 strain untreated or treated with increasing concentrations of various antibiotics and their combination with 2.5 µg/ml 5-FC at 30°C and 37°C was determined. Antibiotics belonging to the class of β-lactams [ceftazidime (CAZ), aztreonam (ATM), and piperacillin (PIP)], aminoglycosides [gentamicin (GEN)], and fluoroquinolones [ciprofloxacin (CIP)] were tested. The untreated preformed biofilm of KTE223 strain is considered 100%. Results of two independent replicates and standard deviations are shown.

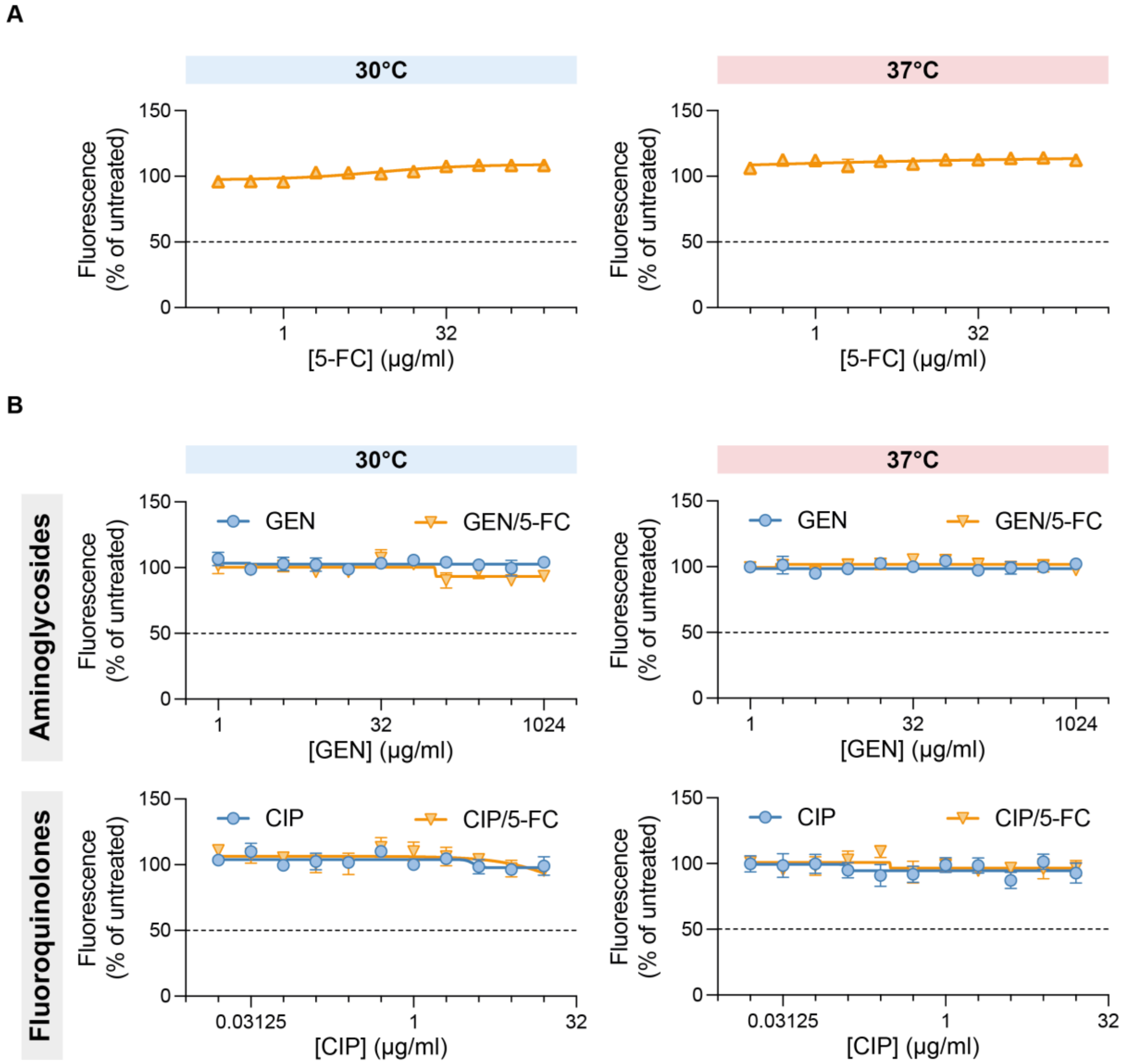

Figure S5. Effect of 5-FC and its combination with aminoglycoside/fluoroquinolone antibiotics on the viability of biofilm-embedded UPEC bacteria. Metabolically active bacterial cells residing in the mature biofilm of the KTE223 strain, untreated or treated with increasing concentrations of various antibiotics and in combination with 2.5 µg/ml 5-FC (5-fluorocytosine) at 30°C and 37°C, were determined. Antibiotics belonging to the class of aminoglycosides [gentamicin (GEN)] and fluoroquinolones [ciprofloxacin (CIP)] were tested. The untreated preformed biofilm of the KTE223 strain is considered to be 100%. Results from two independent replicates and standard deviations are shown.

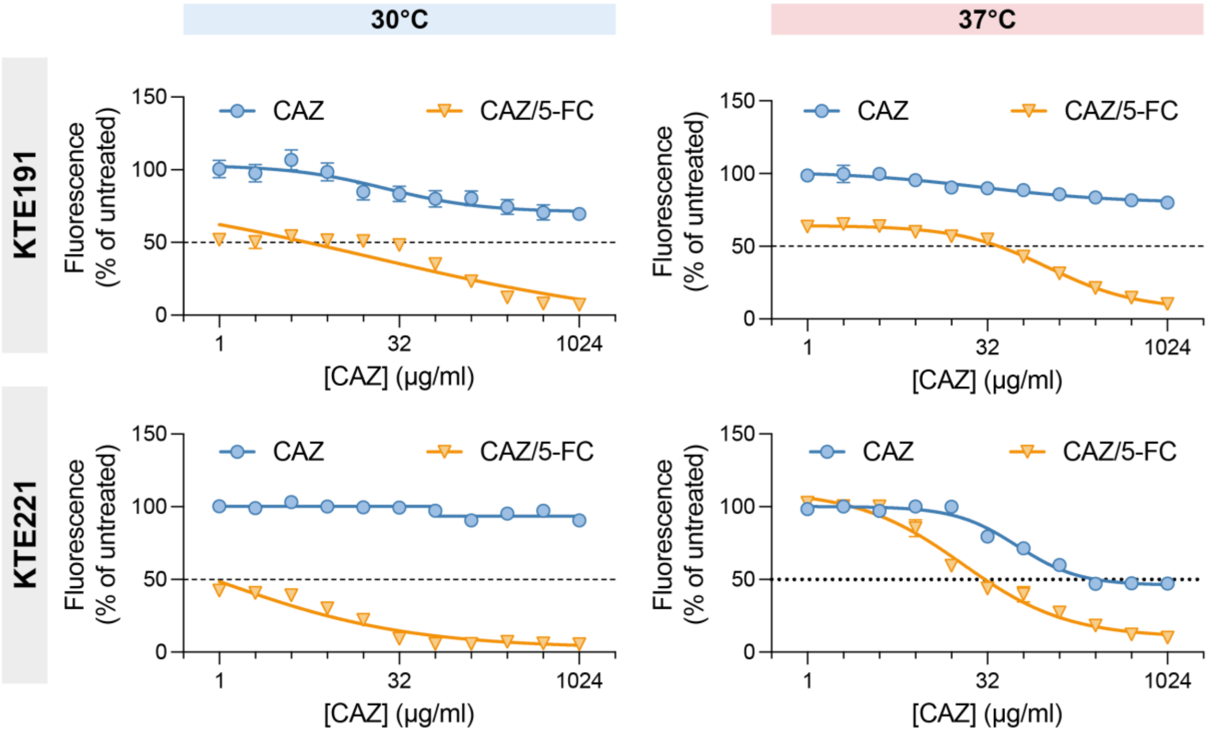

Figure S6. Effect of 5-FC and its combination with ceftazidime antibiotic on the viability of biofilm-embedded UPEC bacteria. Metabolically active bacterial cells residing in the mature biofilm of UPEC strains KTE191 and KTE221, untreated or treated with increasing concentrations of ceftazidime (CAZ) and its combination with 2.5 μg/ml 5-FC (5-fluorocytosine) at 30°C and 37°C, were determined. Untreated preformed biofilm of the tested UPEC strain is considered 100%. Results of two independent replicates and standard deviations are shown.

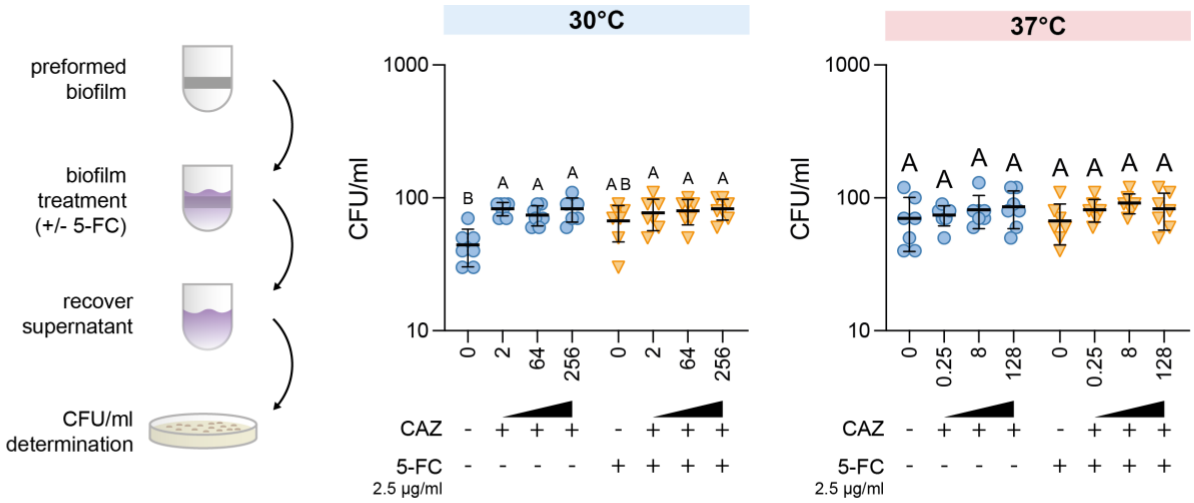

Figure S7. Viability of bacterial cells in spent media after KTE223 mature biofilm treatment with CAZ and its combination with 5-FC. Colony-forming units per ml (CFU/ml) were determined by the agar plating method in spent media recovered after treating KTE223 preformed biofilm with select concentrations of CAZ (ceftazidime) and its combination with 2.5 µg/ml 5-FC (5-fluorocytosine) at 30°C and 37°C. The scheme represents the methodology followed to perform this experiment. Results from 7 independent replicates and standard deviations are shown. Letters indicate significant within-group differences between treatments (One-way ANOVA with Tukey's multiple comparisons test).

Supplementary tables

Table S1. Bacterial strains

Strain	Relevant genotype	Source#	Phylogroup	MLST	Refseq ID	Reference
E. coli K-12 str. MG1655	K-12, F- l- *ilvG*- *rfb*-50 *rph*-1	Laboratory strain	-	-	NC_000913.3	(1)
AM70 (MG1655Δ*csgA*)	MG1655 derivative. Replacement of the *csgA* gene with a chloramphenicol resistance cassette.	Laboratory strain	-	-	-	(2)
PHL628	MG1655 *malA*-Kan *ompR234*	Laboratory strain	-	-	-	(3)
KTE49	Clinical isolate	Fecal, F	B2	131	GCA_000351445.1	(4)
KTE191	Clinical isolate	UTI, F, CA	B2	12*	GCA_000351005.1	(4)
KTE194	Clinical isolate	UTI, F, CA	B2	141	GCA_000352805.1	(4)
KTE207	Clinical isolate	UTI, F, CA	B2	998	GCA_000353005.1	(4)
KTE209	Clinical isolate	UTI, M, CA	B2	73	GCA_000353025.1	(4)
KTE211	Clinical isolate	UTI, F, CA	B2	131	GCA_000353045.1	(4)
KTE216	Clinical isolate	UTI, F, CA	B2	131	GCA_000351225.1	(4)
KTE221	Clinical isolate	UTI, M, HA	A	410	GCA_000408225.1	(4)
KTE223	Clinical isolate	UTI, F, CA	B2	73	GCA_000353125.1	(4)
KTE228	Clinical isolate	UTI, F, CA	D	69	GCA_000351285.1	(4)

*single locus variant

Source: Fecal, isolated from a fecal sample of a healthy donor; UTI, urinary tract infection; M, male patient; F, female patient; CA, community-acquired; HA, hospital-acquired;

Table S2. Primers used for gene expression analysis.

**Name**	**Sequence (5' - 3')**
16S rRNA_RT_frd	TGTCGTCAGCTCGTGTCGTGA
16S rRNA_RT_rev	ATCCCCACCTTCCTCCGGT
csgB_RT_frd	CATAATTGGTCAAGCTGGGACTAA
csgB_RT_rev	GCAACAACCGCCAAAAGTTT
csgD_RT_frd	CCCGTACCGCGACATTG
csgD_RT_rev	AAGGAGGGCTGATTCCGTGCTG
fimA_RT_for	CTCTGGCAATCGTTGTTCTGTC
fimA_RT_rev	TCAACAGAGCCTGCATCAACTG
hlyA_RT_fw	ACTCTCATTCCTGTCCATTGCCGA
hlyA_RT_rev	GAAGCCAGAACAGTGCTTATCGTTGTTA
papC_RT_fw	TACAGTGGCAGTATGAGTAATGACCG
papC_RT_rev	GCGGACTACGATGACTGTAATAGGC
pic_RT_fw	TGTCCGTTCCGATATTGCCTATCAG
pic_RT_rev	GGCCATTGGGGCTTTATCCAGT
sat_RT_fw	TCAATTCCGGATTTTTCTGGTGCAG
sat_RT_rev	AGAAGACTGAGCGTAAACCTGGG
vat_RT_fw	ATCAACGGTTGGTGGCAACAATCC
vat_RT_rev	CCATGGGCGCTTTATCAAGATGTCC
sinH_RT_fw	GACACACTCTCTCCCTACGGTAAGG
sinH_RT_rev	CGTTGCGCAGAAAATTGGCTGAAA
pyrB_RT_frd	CGACAGCGCCAATACATCACT
pyrB_RT_rev	CGGCATTCAGTACCGGTACAT
carA_RT_frd	CGAGAAAGGCGCACAGAATGGC
carA_RT_rev	GGCTTCTGCGGTGGTCACTTCT
fis_RT_frd	AAAACCCCTGCGTGACTCGGTT
fis_RT_rev	CACCATGTCCAACAGGGGCTGT

Supplementary references

1. Blattner FR, Plunkett G, Bloch CA, Perna NT, Burland V, Riley M, Collado-Vides J, Glasner JD, Rode CK, Mayhew GF, Gregor J, Davis NW, Kirkpatrick HA, Goeden MA, Rose DJ, Mau B, Shao Y. 1997. The complete genome sequence of Escherichia coli K-12. Science 277:1453 1462.

2. Tagliabue L, Antoniani D, Maciąg A, Bocci P, Raffaelli N, Landini P. 2010. The diguanylate cyclase YddV controls production of the exopolysaccharide poly-N-acetylglucosamine (PNAG) through regulation of the PNAG biosynthetic pgaABCD operon. Microbiology 156:2901–2911.

3. Vidal O, Longin R, Prigent-Combaret C, Dorel C, Hooreman M, Lejeune P. 1998. Isolation of an Escherichia coli K-12 mutant strain able to form biofilms on inert surfaces: involvement of a new ompR allele that increases curli expression. Journal of Bacteriology 180:2442 2449.

4. Nielsen KL, Stegger M, Kiil K, Godfrey PA, Feldgarden M, Lilje B, Andersen PS, Frimodt-Møller N. 2017. Whole-genome comparison of urinary pathogenic Escherichia coli and faecal isolates of UTI patients and healthy controls. Int J Méd Microbiol 307:497–507.
